# The trends of changes in monitoring indicators related to the risk of recurrent spontaneous abortion

**DOI:** 10.1097/MD.0000000000043604

**Published:** 2025-08-01

**Authors:** Bo Wang, Qian Li, Chunli Li, Shu Li

**Affiliations:** a Department of Obstetrics and Gynecology, Women and Children’s Hospital of Chongqing Medical University, Chongqing, China; b Department of Obstetrics and Gynecology, Chongqing Health Center for Women and Children, Chongqing, China; c Chongqing Research Center for Prevention & Control of Maternal and Child Diseases and Public Health, Chongqing, China; d Department of Clinical Laboratory, Yunyang Maternal and Child Healthcare Hospital, Chongqing, China; e Department of Clinical Laboratory, Women and Children’s Hospital of Chongqing Medical University, Chongqing, China; f Department of Clinical Laboratory, Chongqing Health Center for Women and Children, Chongqing, China.

**Keywords:** anticoagulant proteins, autoantibodies, recurrent spontaneous abortion, thyroid antibodies

## Abstract

The causes of recurrent spontaneous abortion (RSA) are highly complex. A comprehensive analysis of the risk factors for RSA will be beneficial for its diagnosis and treatment. Peripheral blood from RSA patients were collected to test the levels of autoantibodies, thyroid antibodies, anticoagulant proteins, and lupus-anticoagulants. Then, 3 major electronic databases (PubMed, China National Knowledge Infrastructure, and Cochrane Library) were searched to include all detection indicators related to RSA. First, we found that 6 autoantibodies were significantly elevated in RSA patients. Among these, antinuclear antibody (RSA: 32.99 ± 3.85; control group: 13.51 ± 3.30, *P* < .001, *R* = 0.612) showed the highest correlation with RSA. Additionally, the expression levels of anti-double-stranded DNA antibody-IgG antibody (*R* = 0.306) and anti-RA33 IgG antibody (*R* = 0.265) were also highly correlated with RSA. The protein S activity in RSA patients was significantly lower than in the control group. The ratios of lupus-anticoagulants, thyroid peroxidase antibodies, and thyroglobulin antibodies were significantly higher in RSA patients. Then, the meta-analysis revealed that the levels of antinuclear antibody, thyroid peroxidase antibody, anticardiolipid antibody, lupus anticoagulant, DFI, and BMI were significantly elevated in RSA patients, while the activity of protein S and protein C were decreased. Finally, there were no statistically significant differences in the expression levels of these indicators between patients with 2 and 3 miscarriage histories. Given the complexity of the causes of RSA, early multi-indicator combined testing for patients with a history of ≥2 miscarriages can effectively improve the accuracy of diagnosing RSA. This approach can optimize treatment plans and improve the prognosis for patients with RSA.

## 1. Introduction

The causes of recurrent spontaneous abortion (RSA) are highly complex, with a significant portion remaining unknown, and its incidence rate is approximately 3% to 5%.^[1]^ The primary causes of RSA include: chromosomal or genetic abnormalities, anatomical abnormalities (both congenital and acquired), autoimmune diseases, prethrombotic state (including hereditary and acquired), endocrine factors, infections, male factors, and environmental and psychological factors.^[2]^ Even after a comprehensive etiological investigation, about 50% of patients still have unknown causes.^[3]^

Chromosomal abnormalities in embryos can account for 50% to 60% of spontaneous miscarriages, making it the most common cause.^[2]^ The prevalence of uterine malformations in RSA populations is approximately 16%, significantly higher than in the general population.^[4]^ Cervical incompetence typically manifests as painless cervical dilation in the mid to late stages of pregnancy, leading to premature delivery and late miscarriage, representing the primary anatomical factor contributing to late miscarriage.^[5]^ During pregnancy, the immune system undergoes complex changes influenced by hormonal levels, exacerbating local tissue or systemic immune inflammatory damage caused by autoimmune diseases such as antiphospholipid syndrome, systemic lupus erythematosus, Sjogren syndrome (SS), and others.^[4]^ These conditions contribute to endothelial damage, promoting thrombosis, and subsequently affecting placental blood supply and fetal development.^[6]^ Consequently, this can result in adverse pregnancy outcomes such as miscarriage, stillbirth, premature birth, preeclampsia, and fetal growth restriction.^[7]^ Many studies indicate an increased risk of miscarriage associated with thrombophilia, with deficiencies in anticoagulant proteins such as protein C (PC), protein S (PS), and antithrombin-3 being among the most common inherited thrombophilias in Asian countries.^[3,8]^

Considering the complexity and diversity of RSA causes, it is essential to conduct a comprehensive analysis to thoroughly explore factors associated with the risk of RSA. This approach aims to provide support for the prevention and treatment of RSA.

## 2. Materials and methods

### 2.1. Patients

This study included patients with RSA treated at the Chongqing Health Center for Women and Children from January 2017 to December 2023. A control group comprised of patients who had normal deliveries during the same period and had no history of miscarriage was selected. The study received approval from the Ethics Committee of the Chongqing Health Center for Women and Children (2023 ethics department 019), and informed consent was obtained from all subjects during data collection.

### 2.2. Testing autoantibodies

A total of 762 RSA patients and 1213 control patients were included in the study. The iFlash 3000-C chemiluminescence analyzer (YHLO Biotech Co., Ltd., Shenzhen, China) was used for the detection of autoantibodies. The chemiluminescence method was employed to analyze the following antibodies: cytomegalovirus IgG antibody, cytomegalovirus IgM antibody, toxoplasma gondii IgG antibody, toxoplasma gondii IgM antibody, rubella virus IgG antibody, rubella virus IgM antibody, antinuclear antibody (ANA), anti-Müllerian hormone antibody, anti-double-stranded DNA antibody-IgG antibody (dsDNA IgG), anti-SS-A IgG antibody, anti-SS-B IgG antibody, anti-Sm IgG antibody, anti-RNP70 IgG antibody, anti-JO-1 IgG antibody, anti-ScL-70 IgG antibody, anti-RA33 IgG antibody, anti-cyclic citrullinated peptide antibody, rheumatoid factor antibody, anticardiolipid antibody (ACA), ACA-IgG antibody, ACA-IgM antibody, and anti-β2 glycoprotein I antibody.

### 2.3. Testing thyroid function

A total of 1539 RSA patients and 2572 control patients were included in the study. The Abbott Alinity i2000SR automated chemiluminescence immunoassay analyzer (Abbott Laboratories, Abbott Park) was used for the detection of thyroid antibodies. The chemiluminescence method was employed to analyze the expression of thyroglobulin antibody (TgAb) and thyroid peroxidase antibody (TPOAb).

### 2.4. Testing anticoagulant proteins

A total of 704 RSA patients and 662 control patients were included in the study. The Siemens CN-6000 analyzer (Siemens Laboratories, Germany) was used for the detection of anticoagulant proteins. The chromogenic substrate method was employed to detect anticoagulant proteins, including PC and PS, antithrombin 3 (AT-Ⅲ).

### 2.5. Testing lupus anticoagulant

A total of 989 RSA patients and 559 control patients were included in the study. The Siemens CN-6000 analyzer (Siemens Laboratories, Germany) was used for the detection of lupus anticoagulant (LA). The coagulation method was used to detect LA.

### 2.6. Retrieval strategy

For the study, a comprehensive search was conducted across 3 major electronic databases: PubMed, China National Knowledge Infrastructure, and Cochrane Library. The search utilized the following terms: “recurrent spontaneous abortion* or RSA or recurrent pregnancy loss or RPL or recurrent miscarriage* or RM or habitual abortion* or recurrent abortion*.” The search was conducted up to December 2024.

### 2.7. Inclusion and exclusion criteria

Studies were considered eligible for inclusion if they satisfied the following criteria:

(i) The study employed explicit statistical methods; (ii) complete data were available for the study; (iii) the eligible study investigated the risk factors for RSA; (iv) the study has clear diagnostic criteria for RSA; (v) nationality, the language of the literature was not restricted. The race, and age of all patients were also not restricted.

Conversely, studies were excluded if they met the following criteria:

(i) The study had not completed information; (ii) it was a conference report, case report, animal study, cellular study, systematic review, or another type of non-original study; (iii) the study lacked clear grouping methods; (iv) there was no inclusion of a control group.

### 2.8. Screening and data extraction

**Figure 1.**
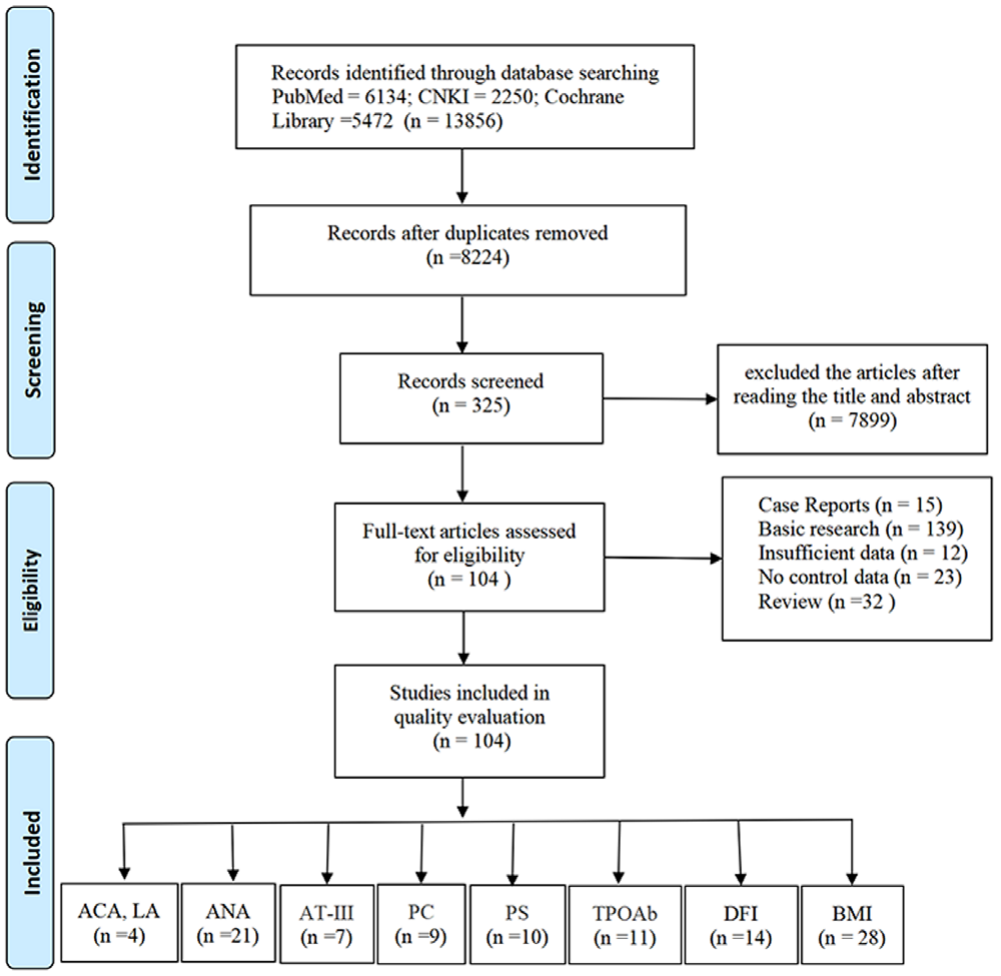
The flow diagram of study selection. ACA = anticardiolipid antibody, ANA = antinuclear antibody, AT-III = antithrombin III, BMI = body mass index, CNKI = China National Knowledge Infrastructure, DFI = DNA fragmentation index, LA = lupus anticoagulant, PC = protein C, PS = protein S, TPOAb = thyroid peroxidase antibody.

The study employed a rigorous process wherein 2 researchers independently reviewed and screened the retrieved studies. In instances of disagreement, comprehensive discussions between the 2 researchers were conducted to reach a consensus. To manage the documents and streamline the screening process, EndNote X7 software (Thomson Corporation, Stamford) was utilized (Fig. 1).

For quality assessment, the Newcastle-Ottawa Scale (NOS) was employed. Each article meeting the inclusion criteria underwent independent evaluation by the researchers. The NOS scoring system categorized articles with a score of <5 as low quality, while those with a score of >6 was considered high quality. Subsequently, articles identified as low quality were excluded from further consideration. This method ensured a thorough and objective evaluation of the selected studies, contributing to the robustness of the study’s findings.

### 2.9. Statistical methods

The SPSS version 25.0 (IBM Corp., Armonk) was used for processing clinical and experimental data. If the data followed a normal distribution, unpaired Student *t* test was employed. Otherwise, nonparametric tests were used. Spearman correlation analysis was used to evaluate the correlation between the risk factors of RSA and RSA. The STATA 12.0 software (Stata Corp LLC, College Station) was used to perform meta-analysis. When there were 2 or more articles examining the association between RSA and the risk factors of RSA, statistical analyses were performed. In cases where there were fewer than 2 relevant articles, descriptive analyses were conducted instead. The Chi-square test and *I*^2^ test were used to assessed heterogeneity among studies. To pool the data, random effect model is adopted. *P* > .05 was considered to have not publication bias.

## 3. Results

### 3.1. Basic information

Patients are divided into 4 groups, and the test results for each group are collected separately. Specific grouping and the number of patients were detailed in Table 1. The age distribution of RSA patients ranges from 19 to 46 years old, while the age distribution of the control group patients ranges from 17 to 52 years old. The age distribution of patients in each group is shown in Table 1.

**Table 1:** The age of included patients.

	Autoantibodies		Thyroid function		Anticoagulant protein		Lupus anticoagulant	
	N	Age	N	Age	N	Age	N	Age
RSA	762	19–45	1539	19–45	704	19–46	989	19–46
Control group	1213	18–52	2572	20–51	662	17–48	559	17–48

N = number of included studies, RSA = recurrent spontaneous abortion.

### 3.2. RSA patients exhibit elevated autoantibodies

We analyzed 22 types of autoantibodies, such as ANA, toxoplasma gondii antibody, rubella virus antibody, dsDNA IgG, and anti-β2 glycoprotein I (β2GPI) antibody. The results revealed that compared to the control group, RSA patients showed significant elevation in 6 types of autoantibodies. Among these, ANA exhibited the highest correlation with RSA. The expression level of ANA in RSA patients was 32.99 ± 3.85, whereas it was 13.51 ± 3.30 in the control group. Correlation analysis indicated a significant positive correlation between ANA and the risk of RSA (*R* = 0.612, *P* < .01). Additionally, the expression levels of dsDNA IgG and anti-RA33 IgG antibody also showed high correlations with RSA, with correlation coefficients of 0.306 and 0.265, respectively (Table 2).

**Table 2:** The expression levels of monitoring indicators in RSA patients.

	RSA			Control group			*P*	*r*	Pr
	Mean	SD	N	Mean	SD	N			
Anti-Tox IgM	0.29	0.01	243	0.28	0.01	1028	.134		
Anti-RV IgM	0.32	0.01	239	0.32	0.01	1005	.957		
Anti-CMV IgM	0.23	0.01	237	0.23	0.01	1013	.716		
Anti-Tox IgG	13.98	3.93	185	11.76	1.68	1019	.606		
Anti-RV IgG	110.70	8.20	220	100.90	4.69	1000	.357		
Anti-CMV IgG	297.10	12.74	179	277.70	8.17	1005	.336		
AMH	2.42	0.16	185	2.42	0.57	18	.993		
ANA	32.99	3.85	365	13.51	3.30	230	**<.001**	0.612	<0.01
dsDNA IgG	5.45	0.47	363	3.36	0.77	229	**.015**	0.306	<0.01
Sm IgG	3.51	0.01	363	3.54	0.04	228	.365		
SS-A IgG	8.13	1.56	365	4.91	1.42	226	.157		
SS-B IgG	7.01	2.27	365	3.59	1.76	226	.286		
RNP70 IgG	4.35	1.29	363	2.00	0.01	224	.228		
JO-1 IgG	2.11	0.06	363	2.17	0.15	224	.710		
ScL-70 IgG	2.13	0.05	363	2.09	0.04	224	.536		
Anti-CCP	0.69	0.06	364	1.20	0.60	225	.291		
RA33 IgG	4.91	1.12	364	2.38	0.11	225	**.025**	0.265	<0.01
RF	3.54	0.79	109	1.99	0.30	34	.281		
ACA	7.03	0.38	149	5.99	0.24	134	**.025**	0.101	0.091
ACA-IgG	2.30	0.16	99	2.38	0.36	49	.820		
ACA-IgM	3.27	0.20	99	2.48	0.12	48	**.011**	0.206	0.012
β2-GPI	4.87	0.46	175	3.66	0.39	120	**.045**	0.083	0.154
PC	105.30	0.70	695	105.70	0.73	662	.651		
PS	88.22	0.69	695	94.73	0.76	662	**<.001**	‐0.17	<0.001
AT-III	96.20	0.38	640	97.55	0.38	554	**.012**	‐0.07	0.011
LA ratio	1.16	0.01	989	1.12	0.01	559	**<.001**	0.17	<0.001
TPOAb	18.81	2.56	1534	13.14	0.62	2571	**.008**	0.09	<0.01
TgAb	55.66	5.90	904	25.9	3.16	748	**<.001**	0.179	<0.01

Statistically significant values are indicated in bold.

ACA = anticardiolipid antibody, ACA-IgG antibody = ACA-IgM antibody, AMH = anti-Müllerian hormone antibody, ANA = antinuclear antibody, anti-CCP = anti-cyclic citrullinated peptide antibody, anti-CMV IgG = cytomegalovirus IgG antibody, anti-CMV IgM = cytomegalovirus IgM antibody, anti-RV IgG = rubella virus IgG antibody, anti-RV IgM = rubella virus IgM antibody, anti-Tox IgG = toxoplasma gondii IgG antibody, anti-Tox IgM = toxoplasma gondii IgM antibody, AT-III = antithrombin 3, dsDNA IgG = anti-double-stranded DNA antibody-IgG antibody, JO-1 IgG = anti-JO-1 IgG antibody, LA = lupus anticoagulant, PC = protein C, Pr = *P* value of related coefficient, PS = protein S, *r* = related coefficient, RA33 IgG = anti-RA33 IgG antibody, RF = rheumatoid factor antibody, RNP70 IgG = anti-RNP70 IgG antibody, RSA = recurrent spontaneous abortion, ScL-70 IgG = anti-ScL-70 IgG antibody, Sm IgG = anti-Sm IgG antibody, SS-A IgG = anti-SS-A IgG antibody, SS-B IgG = anti-SS-B IgG antibody, TgAb = thyroglobulin antibody, TPOAb = thyroid peroxidase antibody, β2-GPI = anti-β2 glycoprotein I antibody.

### 3.3. RSA patients exhibit reduced anticoagulant protein activity

Analysis of anticoagulant protein activity showed that PS activity (88.22 ± 0.69) and AT-III activity (96.20 ± 0.38) in RSA patients were significantly lower compared to the control group (PS: 94.73 ± 0.76; AT-III: 97.55 ± 0.38). PS activity exhibited a negative correlation with the risk of RSA. However, there was no statistically significant difference in PC activity between the 2 groups (Table 2).

### 3.4. RSA patients exhibit elevated levels of lupus anticoagulants and thyroid antibodies

Analysis of lupus anticoagulant expression levels revealed that the LA ratio in RSA patients (1.16 ± 0.01) was significantly higher than in the control group (1.12 ± 0.01). Correlation analysis indicated a positive correlation between LA ratio and the risk of RSA (Table 2).

Additionally, we analyzed the impact of thyroid antibodies on RSA. The results showed that TPOAb (RSA: 18.81 ± 2.56; control group: 13.14 ± 0.63, *P* = .008) and TgAb (RSA: 55.66 ± 5.90; control group: 25.9 ± 3.16, *P* < .001) were significantly higher in RSA patients compared to the control group. Among these, TgAb exhibited a higher correlation with the risk of RSA (Table 2).

### 3.5. Basic characteristics of the included studies

A total of 104 studies meeting the inclusion criteria were ultimately included, yielding 9 indicators related to RSA, including ACA, LA, ANA, AT-Ⅲ, PC, PS, TPOAb, DFI, and BMI. Four studies investigated the relationship between ACA^[4,6,9,10]^ and LA^[4,6,9,10]^ with RSA. Twenty-one studies investigated the relationship between ANA^[3,9,11–25]^ and RSA. Seven studies investigated the relationship between AT-Ⅲ^[8,26–31]^ and RSA. Nine studies investigated the relationship between PC^[8,26–29,31–34]^ and RSA. Ten studies investigated the relationship between PS^[8,26–29,31–35]^ and RSA. Eleven studies investigated the relationship between TPOAb^[17,27,36–44]^ and RSA. Fourteen studies investigated the relationship between DFI^[45–58]^ and RSA. Twenty-eight studies investigated the relationship between BMI^[4,5,7,27,59–79]^ and RSA. All included studies had NOS scores ≥6 (Fig. 1, Tables S1–S9, Supplemental Digital Content, https://links.lww.com/MD/P557).

#### 3.5.1. The levels of ANA, TPOAb, ACA, LA, DFI, and BMI were elevated, while the activities of PS and PC were reduced in RSA patients

**Figure 2.**
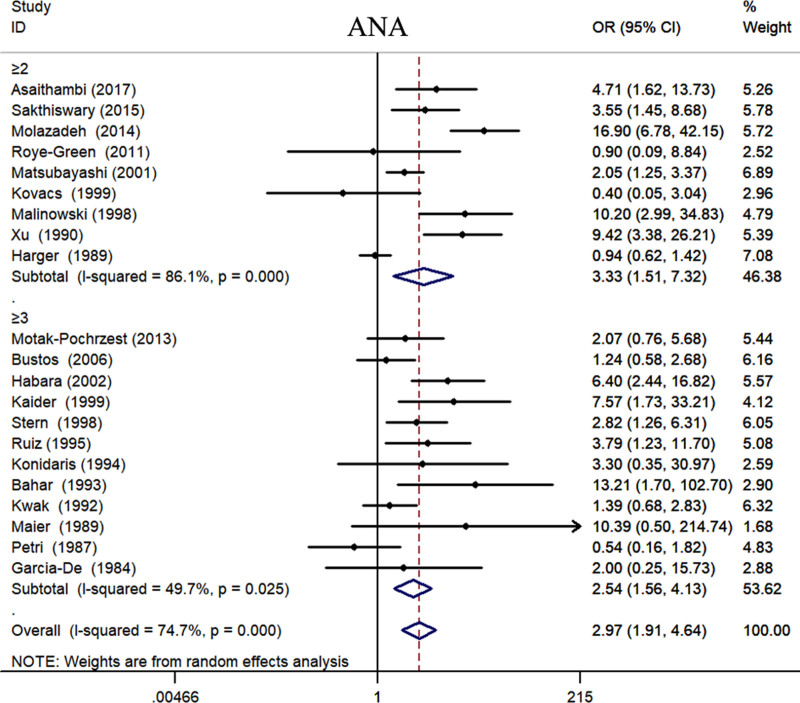
The relationship between the expression level of ANA and the risk of RSA. ANA = antinuclear antibody, CI = confidence interval, OR = odds ratio, RSA = recurrent spontaneous abortion.

**Figure 3.**
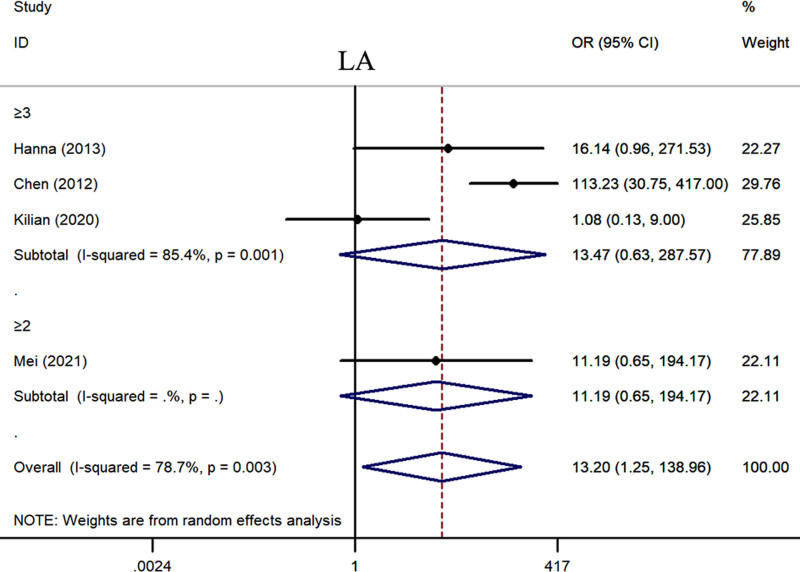
The relationship between the expression level of LA and the risk of RSA. CI = confidence interval, LA = lupus anticoagulant, OR = odds ratio, RSA = recurrent spontaneous abortion.

**Figure 4.**
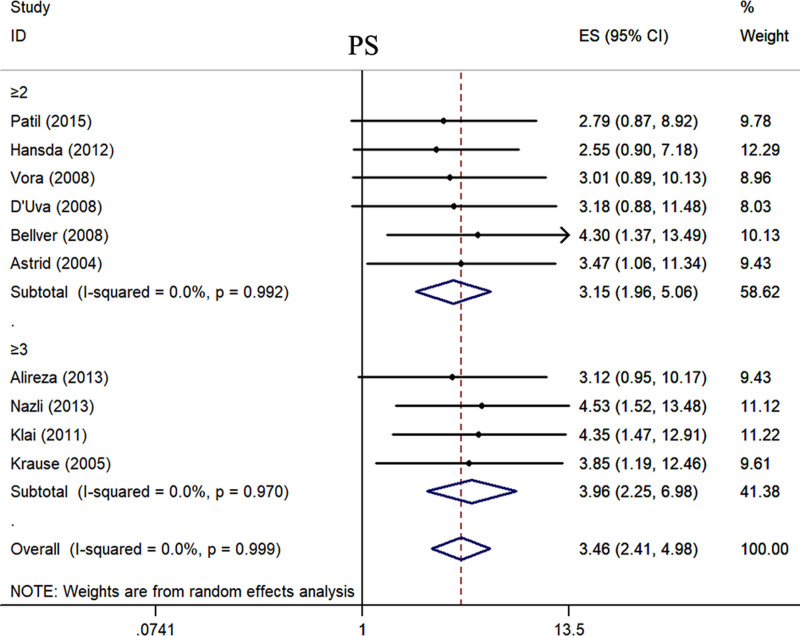
The relationship between the expression level of PS and the risk of RSA. CI = confidence interval, OR = odds ratio, PS = protein S, RSA = recurrent spontaneous abortion.

We separately pooled the data from the included studies according to different indicators. Statistical analysis revealed that the positive rates of ANA (OR = 2.97, 95% CI: 1.91–4.64, *P* < .001) (Fig. 2, Table 3), TPOAb (OR = 1.91, 95% CI: 1.40–2.60, *P* < .001) (Fig. S1, Supplemental Digital Content, https://links.lww.com/MD/P558, Table 3), ACA (OR = 7.32, 95% CI: 1.98–27.03, *P* = .003) (Fig. S2, Supplemental Digital Content, https://links.lww.com/MD/P558, Table 3), and LA (OR = 13.20, 95% CI: 1.25–138.96, *P* = .032) (Fig. 3, Table 3) in patients with RSA were significantly higher than those in the control group. The BMI of RSA patients was 0.34 kg/m² higher than that of the control group (Fig. S3, Supplemental Digital Content, https://links.lww.com/MD/P558, Table 3). We found that the semen DFI rate of the partners of RSA patients was significantly higher than that of the control group (Fig. S4, Supplemental Digital Content, https://links.lww.com/MD/P558, Table 3). We also found that the activities of PC (Fig. S5, Supplemental Digital Content, https://links.lww.com/MD/P558, Table 3) and PS (Fig. 4) were significantly decreased in RSA patients. However, compared with the control group, there was no significant difference in the AT-Ⅲ levels of RSA patients (Fig. S6, Supplemental Digital Content, https://links.lww.com/MD/P558, Table 3).

**Table 3:** The primary outcome pooled through meta-analysis.

Study	N	Z	OR/ES/SMD	95% CI	*P*	*I*^2^ (%)	*P*_H_
ANA	21	4.80	2.97 (OR)	1.91–4.64	<.001	74.7	<0.001
TPOAb	13	4.10	1.91 (OR)	1.40–2.60	<.001	46.1	0.035
ACA	4	2.99	7.32 (OR)	1.98–27.03	.003	0.0	0.626
LA	4	2.15	13.20 (OR)	1.25–138.96	.032	78.7	0.003
DFI	14	7.28	2.39 (SMD)	1.75–3.03	<.001	96.0	<0.001
BMI	26	2.20	0.34 (SMD)	0.04–0.64	.028	97.7	<0.001
PS	10	6.69	3.46 (ES)	2.41–4.98	<.001	0.0	0.999
PC	9	5.37	1.99 (ES)	1.55–2.56	<.001	0.0	0.996
AT-III	7	0.73	0.85 (ES)	0.55–1.32	.467	0.0	0.949

ACA = anticardiolipid antibody, ANA = antinuclear antibody, AT-III = antithrombin III, BMI = body mass index, CI = confidence interval, DFI = DNA fragmentation index, ES = effect size, LA = lupus anticoagulant, OR = odds ratio, PC = protein C, *P*_H_ = *P* of heterogeneity, PS = protein S, SMD = standard mean difference, TPOAb = thyroid peroxidase antibody.

#### 3.5.2. The subgroup analysis by definition of RPL

Currently, there is some controversy regarding the definition of RSA. There is debate on whether RSA should be diagnosed after a history of 2 or more miscarriages or after 3 or more miscarriages. Therefore, our study conducted a stratified analysis of the included studies based on the number of miscarriages. The results revealed that there were no statistically significant differences in the levels of ANA, TPOAb, ACA, LA, DFI, BMI, PS, PC, and AT-Ⅲ between the groups with a history of 2 miscarriages and those with a history of 3 miscarriages. However, it is noteworthy that, although there were no statistically significant differences, the levels of ANA, ACA, LA, DFI, and BMI were higher in patients with more than 2 miscarriages compared to those with more than 3 miscarriages. Additionally, the levels of PS, PC, and AT-Ⅲ were lower in patients with more than 2 miscarriages compared to those with >3 miscarriages. This suggests that screening and treating patients with >2 miscarriages may be more beneficial in improving treatment outcomes and prognosis (Figs. 2–4, Figs. S1–S6, Supplemental Digital Content, https://links.lww.com/MD/P558, Table 4).

**Table 4:** The subgroup analysis by definition of RSA.

Study	Abortive times	N	Z	OR/ES/SMD	95% CI	*P*	*I*^2^ (%)	*P*_H_	*P*_S_
ANA	≥2	9	2.99	3.33 (OR)	1.51–7.32	.003	86.1	<0.001	0.642
	≥3	12	3.75	2.54 (OR)	1.56–4.13	<.001	49.1	0.025	
TPOAb	≥2	6	2.46	1.82 (OR)	1.13–2.92	.014	57.5	0.038	0.184
	≥3	7	3.04	2.03 (OR)	1.29–3.21	.002	42.6	0.107	
ACA	≥2	1	0.17	10.26 (OR)	2.46–42.87	.866	–	–	0.280
	≥3	3	3.19	1.32 (OR)	0.05–32.99	.001	0.0	0.840	
LA	≥2	1	1.66	13.47 (OR)	0.63–287.57	.097	–	–	0.691
	≥3	3	1.66	11.19 (OR)	0.65–194.17	.096	85.4	0.001	
DFI	≥2	7	5.11	3.27 (SMD)	1.83–4.70	<.001	95.9	<0.001	0.633
	≥3	7	4.45	2.00 (SMD)	1.24–3.03	<.001	96.7	<0.001	
BMI	≥2	14	1.34	0.55 (SMD)	0.01–1.09	.179	90.0	<0.001	0.279
	≥3	12	1.98	0.16 (SMD)	‐0.07–0.40	.047	98.8	<0.001	
PS	≥2	6	4.73	3.15 (ES)	1.96–5.06	<.001	0.0	0.992	0.558
	≥3	4	4.77	3.96 (ES)	2.25–6.98	<.001	0.0	0.970	
PC	≥2	5	3.71	1.87 (ES)	1.34–2.61	<.001	0.0	0.990	0.599
	≥3	4	3.91	2.16 (ES)	1.47–3.17	<.001	0.0	0.884	
AT-III	≥2	3	0.95	0.72 (ES)	0.37–1.41	.342	0.0	0.864	0.558
	≥3	4	0.14	0.96 (ES)	0.54–1.71	.887	0.0	0.810	

ACA = anticardiolipid antibody, ANA = antinuclear antibody, AT-III = antithrombin III, BMI = body mass index, CI = confidence interval, DFI = DNA fragmentation index, ES = effect size, LA = lupus anticoagulant, OR = odds ratio, PC = protein C, *P*_H_ = *P* of heterogeneity, PS = protein S, *P*_S_ = *P*-value for comparing definitions of miscarriage, RSA = recurrent spontaneous abortion, SMD = standard mean difference, TPOAb = thyroid peroxidase antibody. “–,” there was only one study, and the heterogeneity could not be calculated.

#### 3.5.3. Publication bias

The Egger linear regression model and Begg funnel plot were used to detect publication bias. The results showed that there was no publication bias in studies on the relationship between ANA, ACA, LA, BMI, PS, and AT-III with RSA (*P* > .05). However, publication bias was found in studies on the relationship between TPOAb (Egger test: *P* = .028), DFI (Begg test: *P* = .001; Egger test: *P* = .008), and PC (Egger test: *P* = .019) with RSA (*P* < .05). The trim-and-fill method was used to adjust this bias, and no studies were added, indicating that the pooled results were stable (Table 5).

**Table 5:** Publication bias.

Study	*P*-value of publication bias		Study	*P*-value of publication bias	
	Begg test	Egger test		Begg test	Egger test
ANA	.085	.083	BMI	.378	.398
TPOAb*	.077	.028	PS	.371	.763
ACA	1.000	.914	PC*	1.000	.019
LA	.734	.104	AT-III	1.000	.288
DFI*	.001	.008			

ACA = anticardiolipid antibody, ANA = antinuclear antibody, AT-III = antithrombin III, BMI = body mass index, DFI = DNA fragmentation index, LA = lupus anticoagulant, PS = protein S, TPOAb = thyroid peroxidase antibody.

* There is publication bias.

## 4. Discussion

The etiology of RSA is highly complex, with many causes remaining unknown.^[6]^ Most of its composition comes from retrospective clinical statistical data, and the proportion of RSA causes changes with the number and timing of previous miscarriages.^[4]^ The main causes of RSA include chromosomal or genetic abnormalities, anatomical abnormalities (congenital and acquired), autoimmune diseases, prethrombotic state, endocrine factors, infections, male factors, environmental factors and psychological factors.^[5]^

In our study, we found that immune factors increase the risk of RSA. Our statistical analysis of clinical data revealed that levels of ANA (32.99 ± 3.85), ACA (7.03 ± 0.38), and LA (1.16 ± 0.01) in RSA patients were significantly higher than those in the control group (ANA: 13.51 ± 3.30, ACA: 5.99 ± 0.24, LA: 1.12 ± 0.01), with statistically significant differences. This conclusion was also confirmed by our meta-analysis. Notably, we found that ANA levels were strongly positively correlated with the risk of recurrent miscarriage (*R* = 0.612). These antibodies can cause decidual vascular lesions and placental thrombosis, leading to fetal ischemia, death, and miscarriage.^[3]^ They also participate in complement activation and placental tissue damage, ultimately resulting in miscarriage.^[11]^ Additionally, these antibodies can directly induce trophoblast cell damage and apoptosis, inhibit the proliferation and differentiation of syncytiotrophoblasts, reduce trophoblast invasion capability, interfere with uterine spiral artery remodeling, and decrease the production of human chorionic gonadotropin and release of growth factors, thereby affecting embryo implantation.^[12]^

Thrombophilia is a pathological process caused by an imbalance in the coagulation and anticoagulation systems due to various factors, including both congenital and acquired causes.^[8]^ Common congenital causes include deficiencies in PC, PS, and AT-III.^[28]^ Thrombophilia during pregnancy can manifest as microthrombosis in uterine spiral arteries or villous vessels, leading to microvascular obstruction in the placenta, affecting uterine–placental blood flow perfusion, and resulting in insufficient blood and oxygen supply.^[34]^ This can cause placental complications such as spontaneous abortion, stillbirth, preeclampsia, fetal growth restriction, and placental abruption.^[34]^ Our analysis of clinical data revealed that the levels of PS and AT-III were significantly lower in recurrent miscarriage patients compared to the control group. This finding is somewhat inconsistent with our meta-analysis results, which showed no difference in AT-III levels between recurrent miscarriage patients and the control group. However, both research approaches indicated that a decrease in PS is associated with a significantly increased risk of recurrent miscarriage.

Some studies have shown that obese women have a significantly higher miscarriage rate compared to women with a normal BMI.^[4,5,7]^ One study analyzed 9587 pregnant women from New Valencia, Madrid, and Barcelona, and found that obese patients had a significantly lower clinical pregnancy rate and live birth rate.^[80]^ These found suggests that obesity is a significant cause of RSA. Our meta-analysis also revealed that the BMI levels in RSA patients are significantly higher compared to the control group. Sperm DNA damage in men is associated with RSA.^[45]^ Bareh et al^[53]^ selected 26 RSA couples and 31 normal fertility couples and used the TUNEL assay to detect sperm DNA fragmentation. The results showed that the DFI rate in the RSA group (36.8%) was significantly higher than in the control group (9.4%). Xiao-Bin et al^[58]^ analyzed 461 RSA couples and 411 normal fertility couples, finding that the DFI rate in the RSA group (42.3%) was significantly higher than in the control group (13.1%). Our meta-analysis also confirmed this conclusion.

Currently, there is still controversy internationally regarding the definition of RSA. According to the guidelines of the American Society for Reproductive Medicine, having a history of 2 or more confirmed spontaneous miscarriages via ultrasound or histological examination is sufficient to diagnose RSA.^[81]^ The Royal College of Obstetricians and Gynaecologists in the UK defines RSA as 3 or more consecutive pregnancy failures before 24 weeks of gestation with the same partner.^[77]^ Given this controversy, we performed a stratified analysis based on the diagnostic definitions of RSA, and the results indicated that there were no statistically significant differences in the monitoring indicators for recurrent miscarriage. Therefore, screening for causes in RSA populations with a history of 2 or more miscarriages, and providing interventions before a subsequent pregnancy for women with a history of 2 miscarriages, can effectively improve clinical treatment outcomes and prognosis for RSA patients.

In conclusion, the etiology of RSA is complex, with many cases remaining unexplained. This complexity increases the difficulty of treatment and the economic burden of RSA patients. Early multiparameter screening in patients with a history of 2 or more miscarriages can effectively improve the accuracy of RSA diagnosis.

## Author contributions

**Conceptualization:** Shu Li, Chunli Li.

**Data curation:** Qian Li.

**Formal analysis:** Bo Wang.

**Methodology:** Shu Li, Chunli Li.

**Software:** Shu Li, Bo Wang.

**Visualization:** Shu Li.

**Writing – original draft:** Shu Li, Bo Wang, Chunli Li.

**Writing – review & editing:** Shu Li, Bo Wang, Chunli Li.

## Supplementary Material


