# Supplementary material for: The trends of changes in monitoring indicators related to the risk of recurrent spontaneous abortion
Source: Medicine (Baltimore). 2025 Aug 1;104(31):e43604. doi: 10.1097/MD.0000000000043604 (PMC12324057; doi:10.1097/MD.0000000000043604)
Supplement: Supplementary file 1 [file medi-104-e43604-s001.docx]

Supplementary table1. The main characteristics of the included studies on the relationship between LA and RSA.

| Author | Year | Country | RSA | | | | Control group | | | | Assay | NOS |
| --- | --- | --- | --- | --- | --- | --- | --- | --- | --- | --- | --- | --- |
|  |  |  | + | - | Total | Losses | + | - | Total | Live birth |  |  |
| Hanna | 2013 | Poland | 21 | 134 | 155 | ≥3 | 0 | 50 | 50 | ≥1 | DRVVT | 8 |
| Chen | 2012 | China | 96 | 13 | 109 | ≥3 | 3 | 46 | 49 | ≥1 | DRVVT | 7 |
| Kilian | 2020 | Austria | 6 | 759 | 765 | ≥3 | 1 | 136 | 137 | ≥1 | DRVVT | 8 |
| Mei | 2021 | China | 11 | 93 | 104 | ≥2 | 0 | 45 | 45 | ≥1 | DRVVT | 7 |

DRVVT, dilute Russell Viper Venom test. LA, lupus anti-coagulant. NOS, Newcastle - Ottawa Scale. RSA, recurrent spontaneous abortion.

Supplementary table 2. The main characteristics of the included studies on the relationship between ACA and RSA.

| Author | Year | Country | RSA | | | | Control group | | | | Assay | NOS |
| --- | --- | --- | --- | --- | --- | --- | --- | --- | --- | --- | --- | --- |
|  |  |  | + | - | Total | Losses | + | - | Total | Live birth |  |  |
| Hanna | 2013 | Poland | 25 | 130 | 155 | ≥3 | 0 | 50 | 50 | ≥1 | ELISA | 8 |
| Chen | 2012 | China | 14 | 95 | 109 | ≥3 | 1 | 48 | 49 | ≥1 | ELISA | 7 |
| Kilian | 2020 | Austria | 26 | 685 | 711 | ≥3 | 0 | 138 | 138 | ≥1 | ELISA | 8 |
| Mei | 2021 | China | 1 | 103 | 104 | ≥2 | 0 | 45 | 45 | ≥1 | ELISA | 7 |

ELISA, enzyme linked immunosorbent assay. ACA, anticardiolipid antibody. NOS, Newcastle - Ottawa Scale. RSA, recurrent spontaneous abortion.

Supplementary table 3. The main characteristics of the included studies on the relationship between ANA and RSA.

| Author | Year | Country | RSA | | | | Control group | | | | Assay | NOS |
| --- | --- | --- | --- | --- | --- | --- | --- | --- | --- | --- | --- | --- |
|  |  |  | + | - | Total | Losses | + | - | Total | Live birth |  |  |
| Asaithambi | 2017 | India | 18 | 42 | 60 | ≥2 | 5 | 55 | 60 | ≥1 | IIF | 7 |
| Sakthiswary | 2015 | Malaysia | 24 | 44 | 68 | ≥2 | 8 | 52 | 60 | ≥1 | IIF | 7 |
| Molazadeh | 2014 | Iran | 74 | 486 | 560 | ≥2 | 5 | 555 | 560 | Healthy controls | IIF | 9 |
| Motak-Pochrzest | 2013 | Poland | 29 | 126 | 155 | ≥3 | 5 | 45 | 50 | Healthy controls | IIF | 7 |
| Roye-Green | 2011 | Jamaica | 1 | 49 | 50 | ≥2 | 3 | 132 | 135 | ≥1 | IIF | 8 |
| Bustos | 2006 | Argentina | 16 | 102 | 118 | ≥3 | 14 | 111 | 125 | ≥1 | IIF | 7 |
| Habara | 2002 | Japan | 20 | 29 | 49 | ≥3 | 7 | 65 | 72 | Healthy controls | IIF | 9 |
| Matsubayashi | 2001 | Japan | 64 | 209 | 273 | ≥2 | 26 | 174 | 200 | Healthy controls | IIF | 8 |
| Kaider | 1999 | USA | 138 | 164 | 302 | ≥3 | 2 | 18 | 20 | Healthy controls | ELISA | 8 |
| Kovacs | 1999 | Hungary | 2 | 57 | 59 | ≥2 | 2 | 23 | 25 | ≥1 | IIF | 9 |
| Stern | 1998 | New Zealand | 22 | 75 | 97 | ≥3 | 10 | 96 | 106 | Healthy controls | IIF | 7 |
| Malinowski | 1998 | Poland | 50 | 67 | 117 | ≥2 | 3 | 41 | 44 | ≥1 | IIF | 7 |
| Ruiz | 1995 | USA | 21 | 72 | 93 | ≥3 | 4 | 52 | 56 | ≥1 | IIF | 7 |
| Konidaris | 1994 | Greece | 4 | 40 | 44 | ≥3 | 1 | 33 | 34 | Healthy controls | IIF | 7 |
| Bahar | 1993 | Kuwait | 14 | 89 | 103 | ≥3 | 1 | 84 | 85 | ≥1 | IIF | 7 |
| Kwak | 1992 | USA | 29 | 124 | 153 | ≥3 | 13 | 77 | 90 | ≥1 | IIF | 7 |
| Xu | 1990 | USA | 12 | 18 | 30 | ≥2 | 8 | 113 | 121 | Healthy controls | IIF | 7 |
| Harger | 1989 | USA | 43 | 232 | 275 | ≥2 | 69 | 349 | 418 | Healthy controls | IIF | 8 |
| Maier | 1989 | USA | 3 | 16 | 19 | ≥3 | 0 | 24 | 24 | Healthy controls | IIF | 7 |
| Petri | 1987 | USA | 5 | 37 | 42 | ≥3 | 8 | 32 | 40 | Healthy controls | IIF | 7 |
| Garcia-De | 1984 | Mexico | 2 | 14 | 16 | ≥3 | 2 | 28 | 30 | ≥1 | IIF | 7 |

IIF, indirect immunoﬂuorescence. ELISA, enzyme linked immunosorbent assay. ANA, antinuclear antibody. NOS, Newcastle - Ottawa Scale. RSA, recurrent spontaneous abortion.

Supplementary table 4. The main characteristics of the included studies on the relationship between AT-Ⅲ and RSA.

| Author | Year | Country | Losses | OR | LCI | UCI | P | Total | NOS |
| --- | --- | --- | --- | --- | --- | --- | --- | --- | --- |
| Patil | 2015 | India | ≥2 | 0.6 | 0.19 | 1.84 | 0.37 | 678 | 9 |
| [Nazli](https://pubmed.ncbi.nlm.nih.gov/?sort=pubdate&term=Hossain+N&cauthor_id=22690883) | 2013 | Pakistan | ≥3 | 1.51 | 0.5 | 4.5 | 0.463 | 321 | 8 |
| Klai | 2011 | Tunisia | ≥3 | 0.89 | 0.27 | 2.94 | 0.846 | 163 | 7 |
| Ayadurai | 2009 | Malaysia | ≥3 | 0.81 | 0.24 | 2.69 | 0.728 | 562 | 8 |
| Vora | 2008 | India | ≥2 | 0.68 | 0.21 | 2.27 | 0.535 | 298 | 7 |
| Bellver | 2008 | Spain | ≥2 | 0.93 | 0.29 | 3 | 0.9 | 62 | 6 |
| Krause | 2005 | Germany | ≥3 | 0.73 | 0.23 | 2.27 | 0.582 | 266 | 7 |

OR, odds ratio. LCI, lower confidence interval. UCI, upper confidence interval. NOS, Newcastle - Ottawa Scale. RSA, recurrent spontaneous abortion. AT-Ⅲ, antithrombin Ⅲ.

Supplementary table 5. The main characteristics of the included studies on the relationship between PC and RSA.

| Author | Year | Country | Losses | OR | LCI | UCI | P | Total | NOS |
| --- | --- | --- | --- | --- | --- | --- | --- | --- | --- |
| Patil | 2015 | India | ≥2 | 1.68 | 0.79 | 3.6 | 0.181 | 678 | 9 |
| Parand | 2013 | Iran | ≥3 | 1.99 | 0.94 | 4.21 | 0.073 | 134 | 8 |
| [Nazli](https://pubmed.ncbi.nlm.nih.gov/?sort=pubdate&term=Hossain+N&cauthor_id=22690883) | 2013 | Pakistan | ≥3 | 2.96 | 1.25 | 7 | 0.014 | 321 | 8 |
| Hansda | 2012 | India | ≥2 | 1.72 | 0.83 | 3.59 | 0.147 | 100 | 7 |
| Klai | 2011 | Tunisia | ≥3 | 2.05 | 0.96 | 4.4 | 0.064 | 163 | 7 |
| Vora | 2008 | India | >2 | 1.88 | 0.89 | 4 | 0.1 | 298 | 7 |
| D'Uva | 2008 | Italy | ≥2 | 1.93 | 0.93 | 4.01 | 0.078 | 190 | 8 |
| Bellver | 2008 | Spain | ≥2 | 2.17 | 1.05 | 4.51 | 0.037 | 62 | 6 |
| Krause | 2005 | Germany | ≥3 | 1.95 | 0.94 | 4.04 | 0.073 | 266 | 7 |

OR, odds ratio. LCI, lower confidence interval. UCI, upper confidence interval. NOS, Newcastle - Ottawa Scale. RSA, recurrent spontaneous abortion. PC, protein C.

Supplementary table 6. The main characteristics of the included studies on the relationship between PS and RSA.

| Author | Year | Country | Losses | OR | LCI | UCI | P | Total | NOS |
| --- | --- | --- | --- | --- | --- | --- | --- | --- | --- |
| Patil | 2015 | India | ≥2 | 2.79 | 0.87 | 8.92 | 0.084 | 678 | 9 |
| Alireza | 2013 | Iran | ≥3 | 3.12 | 0.95 | 10.17 | 0.06 | 134 | 8 |
| [Nazli](https://pubmed.ncbi.nlm.nih.gov/?sort=pubdate&term=Hossain+N&cauthor_id=22690883) | 2013 | Pakistan | ≥3 | 4.53 | 1.52 | 13.48 | 0.007 | 321 | 8 |
| Hansda | 2012 | India | ≥2 | 2.55 | 0.9 | 7.18 | 0.077 | 100 | 7 |
| Klai | 2011 | Tunisia | ≥3 | 4.35 | 1.47 | 12.91 | 0.008 | 163 | 7 |
| Vora | 2008 | India | ≥2 | 3.01 | 0.89 | 10.13 | 0.075 | 298 | 7 |
| D'Uva | 2008 | Italy | ≥2 | 3.18 | 0.88 | 11.48 | 0.077 | 190 | 8 |
| Bellver | 2008 | Spain | ≥2 | 4.3 | 1.37 | 13.49 | 0.013 | 62 | 6 |
| Krause | 2005 | Germany | ≥3 | 3.85 | 1.19 | 12.46 | 0.024 | 266 | 7 |
| Astrid | 2004 | Austria | ≥2 | 3.47 | 1.06 | 11.34 | 0.04 | 97 | 7 |

OR, odds ratio. LCI, lower confidence interval. UCI, upper confidence interval. NOS, Newcastle - Ottawa Scale. RSA, recurrent spontaneous abortion. PS, protein S.

Supplementary table 7. The main characteristics of the included studies on the relationship between TPOAb and RSA.

| Author | Year | Country | RSA | | | | Control group | | | | Assay | NOS |
| --- | --- | --- | --- | --- | --- | --- | --- | --- | --- | --- | --- | --- |
|  |  |  | + | - | Total | Losses | + | - | Total | Live birth |  |  |
| Bagis（2） | 2001 | Turkey | 3 | 78 | 81 | ≥3 | 3 | 792 | 795 | ≥1 | CLEIA | 8 |
| Bussen | 1995 | Germany | 5 | 1 | 6 | ≥3 | 17 | 21 | 38 | ≥1 | ELISA | 8 |
| Bussen | 1997 | Germany | 6 | 1 | 7 | ≥3 | 22 | 27 | 49 | ≥1 | ELISA | 7 |
| Iravani | 2008 | Iran | 117 | 28 | 145 | ≥3 | 524 | 241 | 765 | ≥1 | ELISA | 6 |
| Kaider | 1999 | USA | 47 | 14 | 61 | ≥3 | 255 | 82 | 337 | ≥1 | SGAA | 7 |
| Pratt | 1993 | USA | 9 | 16 | 25 | ≥3 | 36 | 84 | 120 | ≥1 | KRIA | 7 |
| Ticconi（2） | 2011 | Italy | 16 | 23 | 39 | ≥3 | 52 | 169 | 221 | ≥1 | CLIA | 7 |
| Bagis（1） | 2001 | Turkey | 9 | 72 | 81 | ≥2 | 24 | 771 | 795 | ≥1 | CLEIA | 8 |
| Bellver | 2008 | Spain | 0 | 4 | 4 | ≥2 | 30 | 28 | 58 | ≥1 | TIA | 6 |
| Kutteh | 1999 | USA | 104 | 22 | 126 | ≥2 | 596 | 178 | 774 | ≥1 | ELISA | 8 |
| Lata | 2013 | India | 31 | 18 | 49 | ≥2 | 69 | 82 | 151 | ≥1 | ECLIA | 8 |
| Mumusoglu | 2015 | Turkey | 17 | 50 | 67 | ≥2 | 102 | 346 | 448 | ≥1 | RIA | 6 |
| Ticconi（1） | 2011 | Italy | 31 | 8 | 39 | ≥2 | 129 | 92 | 221 | ≥1 | CLIA | 7 |

CLIA, chemiluminescent immunoassay. ELISA, enzyme-linked immuno sorbent assay. RIA, radioimmunoassay. TIA, two-site immunoluminometric assay. CLEIA, chemiluminescent enzyme immunometric assay. KRIA, kalibre radioimmunoassay. SGAA, serodia gel-agglutination assay. ECLIA, electro-chemiluminescence immunoassay. NOS, Newcastle - Ottawa Scale. RSA, recurrent spontaneous abortion. TPOAb anti-thyroperoxidase antibody.

Supplementary table 8. The main characteristics of the included studies on the relationship between DFI and RSA.

| Author | Year | Country | RSA | | | | Control group | | | | Assay | NOS |
| --- | --- | --- | --- | --- | --- | --- | --- | --- | --- | --- | --- | --- |
|  |  |  | Mean | SD | Total | Losses | Mean | SD | Total | Live birth |  |  |
| Esquerre-Lamare | 2018 | France | 6.8 | 5.6 | 33 | ≥3 | 6.6 | 5.5 | 27 | ≥1 | SCSA | 7 |
| Zidi-Jrah | 2016 | Tunisia | 17.1 | 9.3 | 22 | ≥2 | 11.8 | 5.7 | 20 | ≥1 | TUNEL | 7 |
| Bareh | 2016 | USA | 36.8 | 2.7 | 26 | ≥2 | 9.4 | 2.7 | 31 | ≥1 | TUNEL | 7 |
| Coughlan | 2015 | UK | 10.9 | 1.9 | 16 | ≥3 | 7.2 | 3.7 | 7 | ≥1 | SCD | 8 |
| Ruixue | 2013 | China | 25.6 | 11.5 | 68 | ≥3 | 20 | 7.7 | 63 | ≥1 | Aniline blue | 7 |
| Ribas | 2012 | Spain | 19.3 | 6.1 | 20 | ≥2 | 12.2 | 4.6 | 25 | ≥1 | SCD | 8 |
| Kumar | 2012 | India | 28.1 | 5 | 45 | ≥3 | 21.8 | 4.8 | 20 | ≥1 | SCSA | 7 |
| Zhang | 2012 | China | 15.2 | 6.4 | 111 | ≥2 | 13.9 | 4.4 | 30 | ≥1 | SCD | 7 |
| Absalan | 2012 | Iran | 23.3 | 1.1 | 30 | ≥3 | 11.6 | 0.5 | 30 | ≥1 | SCD | 7 |
| Imam | 2011 | India | 23.4 | 9.9 | 20 | ≥3 | 13.9 | 5.4 | 20 | ≥1 | SCSA | 8 |
| Brahem | 2011 | Tunisia | 32.2 | 6.1 | 31 | ≥2 | 10.2 | 2.1 | 20 | ≥1 | TUNEL | 7 |
| Carrell | 2003 | USA | 38.3 | 4.2 | 21 | ≥3 | 11.9 | 1 | 26 | ≥1 | SCD | 7 |
| Tania | 2017 | Italy | 18.8 | 7 | 112 | ≥2 | 12.8 | 5.3 | 114 | ≥1 | TUNEL | 8 |
| Xiao-bin | 2020 | China | 25.88 | 12.58 | 461 | ≥2 | 17.09 | 10.08 | 411 | ≥1 | SCSA | 8 |

SCD, sperm chromatin dispersion test. SCSA, sperm chromatin structure assay. TUNEL, terminal deoxynucleotide transferase - mediated dUTP nick-end labeling assay. NOS, Newcastle - Ottawa Scale. RSA, recurrent spontaneous abortion. DFI, DNA fragmentation index.

Supplementary table 9. The main characteristics of the included studies on the relationship between BMI and RSA.

| Author | Year | Country | RSA | | | | Control group | | | | NOS |
| --- | --- | --- | --- | --- | --- | --- | --- | --- | --- | --- | --- |
|  |  |  | Mean | SD | Total | Losses | Mean | SD | Total | Live birth |  |
| Ahmed | 2015 | Bahrain | 26.3 | 5.4 | 275 | ≥3 | 25.2 | 4.3 | 290 | Healthy controls | 8 |
| Almawi | 2013 | Bahrain | 26.3 | 5.4 | 296 | ≥3 | 25.2 | 4.3 | 305 | Healthy controls | 9 |
| Al-Shaikh | 2013 | Bahrain | 26.3 | 5.4 | 287 | ≥3 | 25.1 | 4.3 | 308 | Healthy controls | 8 |
| Bahia | 2018 | Tunisia | 25.5 | 4.1 | 396 | ≥3 | 26.5 | 5.5 | 361 | ≥2 | 9 |
| Bennett | 2014 | UK | 24.7 | 4.3 | 50 | ≥3 | 24.2 | 4.6 | 41 | Healthy controls | 6 |
| Bussen | 1999 | Germany | 26.1 | 6.4 | 42 | ≥3 | 22.6 | 3 | 42 | Healthy controls | 7 |
| Cao | 2013 | China | 20.9 | 2.2 | 94 | ≥2 | 20.1 | 2.2 | 169 | ≥1 | 8 |
| Chin | 2013 | USA | 26.4 | 7.2 | 99 | ≥2 | 26.9 | 7.1 | 108 | ≥2 | 8 |
| Comba | 2015 | Turkey | 24.5 | 2.3 | 21 | ≥2 | 25.1 | 3.1 | 20 | Healthy controls | 6 |
| Dundar | 2015 | Turkey | 22.9 | 3.4 | 60 | ≥3 | 22.4 | 3.1 | 60 | Healthy controls | 7 |
| Ayla | 2016 | Turkey | 25.5 | 3.5 | 42 | ≥2 | 22.8 | 4 | 36 | Healthy controls | 6 |
| Michaela | 2012 | Sweden | 24.7 | 4.8 | 188 | ≥3 | 23.9 | 4 | 391 | ≥2 | 9 |
| Ispasoiu | 2013 | Romania | 26.2 | 4.7 | 65 | ≥2 | 25.6 | 4.8 | 53 | ≥1 | 7 |
| Yonghui (1) | 2016 | China | 23.2 | 2.9 | 154 | ≥3 | 22.8 | 2.7 | 155 | Healthy controls | 8 |
| Yonghui (2) | 2016 | China | 22.5 | 4.4 | 152 | ≥3 | 21.6 | 3 | 151 | Healthy controls | 8 |
| Lan | 2018 | China | 24.3 | 3.3 | 129 | ≥2 | 22.4 | 2.7 | 116 | Healthy controls | 8 |
| [Shang](https://pubmed.ncbi.nlm.nih.gov/?sort=pubdate&term=Li+S&cauthor_id=29163762) | 2017 | China | 20.3 | 1.3 | 80 | ≥2 | 20.4 | 1.1 | 100 | ≥1 | 7 |
| Park | 2019 | Korea | 21.6 | 3.7 | 375 | ≥2 | 21.6 | 3.2 | 276 | Healthy controls | 9 |
| Pekcan | 2017 | Turkey | 24.6 | 2.8 | 45 | ≥2 | 25.7 | 4 | 41 | ≥2 | 7 |
| Romero | 2016 | USA | 26 | 6.4 | 117 | ≥2 | 26.6 | 5.8 | 117 | ≥1 | 8 |
| Sater | 2012 | Bahrain | 26 | 5.1 | 277 | ≥3 | 24.8 | 3.9 | 288 | Healthy controls | 8 |
| [Rita](https://pubmed.ncbi.nlm.nih.gov/?sort=pubdate&term=Sharshiner+R&cauthor_id=24176553) | 2013 | USA | 26.4 | 6.7 | 116 | ≥2 | 26.5 | 6.1 | 116 | ≥1 | 8 |
| Trifonova | 2019 | Russia | 23.8 | 4.7 | 253 | ≥2 | 24.2 | 4.1 | 339 | ≥2 | 8 |
| Zahraei | 2014 | Iran | 29.3 | 5.2 | 100 | ≥2 | 22.8 | 2.7 | 100 | Healthy controls | 7 |
| Bellver | 2008 | Spain | 22.7 | 2.5 | 30 | ≥2 | 23.8 | 3.5 | 32 | Healthy controls | 7 |
| Kilian | 2020 | Austria | 34.24 | 4.59 | 820 | ≥3 | 15.56 | 1.92 | 141 | Healthy controls | 8 |

BMI, body mass index. NOS, Newcastle - Ottawa Scale. RSA, recurrent spontaneous abortion.
